# Quantitative Microbial Risk Assessment of Drinking Water Quality to Predict the Risk of Waterborne Diseases in Primary-School Children

**DOI:** 10.3390/ijerph17082774

**Published:** 2020-04-17

**Authors:** Jamil Ahmed, Li Ping Wong, Yan Piaw Chua, Najeebullah Channa, Rasool Bux Mahar, Aneela Yasmin, James A. VanDerslice, Joshua V. Garn

**Affiliations:** 1 Centre for Epidemiology and Evidence-Based Practice, Department of Social and Preventive Medicine, Faculty of Medicine, University of Malaya, Kuala Lumpur 50603, Malaysia; 2 US- Pakistan Center for Advanced Studies in Water, Mehran University of Engineering & Technology, Jamhsoro 76062, Pakistan; najeebchanna@outlook.com (N.C.); rb.mahar@faculty.muet.edu.pk (R.B.M.); 3 Institute of Educational Leadership, Level 11, Wisma R & D, UM, University of Malaya, Jalan Pantai Baru, Kuala Lumpur 59000, Malaysia; chuayp@um.edu.my; 4 Department of Biotechnology, Sindh Agriculture University, Tandojam 70060, Sindh, Pakistan; aneelayasmin@yahoo.com; 5 Department of Family and Preventive Medicine, University of Utah, Salt Lake City, UT 84112, USA; jim.vanderslice@utah.edu; 6 School of Community Health Sciences, University of Nevada, Reno, NV 89557, USA; jgarn@unr.edu

**Keywords:** QMRA, water quality, pathogens, health-risk assessment, primary-school children

## Abstract

Primary-school children in low- and middle-income countries are often deprived of microbiologically safe water and sanitation, often resulting in a high prevalence of gastrointestinal diseases and poor school performance. We used Quantitative Microbial Risk Assessment (QMRA) to predict the probability of infection in schoolchildren due to consumption of unsafe school water. A multistage random-sampling technique was used to randomly select 425 primary schools from ten districts of Sindh, Pakistan, to produce a representative sample of the province. We characterized water supplies in selected schools. Microbiological testing of water resulted in inputs for the QMRA model, to estimate the risks of infections to schoolchildren. Groundwater (62%) and surface water (38%) were identified as two major sources of drinking water in the selected schools, presenting varying degrees of health risks. Around half of the drinking-water samples were contaminated with *Escherichia coli* (49%), *Shigella* spp. (63%), *Salmonella* spp. (53%), and *Vibrio cholerae* (49%). Southern Sindh was found to have the highest risk of infection and illness from *Campylobacter* and *Rotavirus*. Central and Northern Sindh had a comparatively lower risk of waterborne diseases. Schoolchildren of Karachi were estimated to have the highest probability of illness per year, due to *Campylobacter* (70%) and *Rotavirus* (22.6%). Pearson correlation was run to assess the relationship between selected pathogens. *V. cholerae* was correlated with *Salmonella* spp., *Campylobacter*, *Rotavirus*, and *Salmonella* spp. Overall, the risk of illness due to the bacterial infection (*E. coli, Salmonella* spp., *V. cholerae*, *Shigella*, and *Campylobacter*) was high. There is a dire need for management plans in the schools of Sindh, to halt the progression of waterborne diseases in school-going children.

## 1. Introduction

The United Nations has estimated that at least 2.5 billion people in low- and middle-income countries lack access to improved sanitation, and over 884 million lack access to improved drinking water [1]. As a result, an estimated 2.3 billion people across the world are suffering from water-related diseases [2]. Consumption of pathogenic microorganisms in drinking water may pose serious health risks due to waterborne diseases [3,4]. Drinking water is considered one of the important reservoirs of microbes in developing countries. Four Billion cases of diarrhea are reported each year, of which 2.2 million result in death, and children under five years are the main victims. Furthermore, more than 90% of the disease burden is contributed by the developing world [5,6]. Each bout of diarrhea progressively compromises the immune system, indirectly killing many more millions each year [7].

In Pakistan, rapid population growth, industrialization, and urbanization have stressed the water resources and compromised water quality, which subsequently negatively impacts children’s school performance [8,9,10]. A recent study revealed that 21 million people in Pakistan are deprived of basic access to safe water [11]. Furthermore, Pakistan is among the top ten countries in the world with the lowest access to clean and safe water close to the home [11]. Water from unprotected springs and wells add to the high morbidity and mortality rate in Pakistan [12]. Main water sources are often contaminated with various pathogenic bacteria and hazardous chemicals, coming from the untreated discharge of influents into water bodies [13]. Waterborne diseases due to unimproved water include diarrhea, typhoid, dysentery, cholera, and Hepatitis A, which are leading causes of morbidity and mortality among children in Pakistan [12,14,15].

One of the serious impacts of diarrheal morbidity among children is the loss of school time due to illness. One study estimated that 670,000 Pakistani children miss school on average per day, due to water-related diseases [10]. Pakistani schools often have inadequate access to and use of water facilities. According to Sindh Education Management Information System (SEMIS), most of the primary schools of Sindh, Pakistan, do not have a safe drinking-water source, and the majority of schools are in need of clean drinking-water systems [9,10]. Schools often lack water-storage capacity; therefore, they must use alternate water sources, such as private venders, local wells, and reservoirs (springs and rainwater storage points). Local vendors are often unregistered, with no checks on them by authorities, and unhygienic handling of these sources may lead to contamination [15]. One local study in schools showed that only 25% of schools had onsite treatment facilities (mainly UV light) [8]. In another study in Pakistan, only one out of 14 water-filtration plants were meeting national water-quality standards. Both of these studies recommended periodic water-quality monitoring from national water-quality-monitoring authorities, to ensure safe drinking water at the point of use [8,16].

Quantitative Microbial Risk Assessment (QMRA) is a tool to estimate the risk of an adverse effect, such as illness, infection, and/or death from exposure to waterborne, airborne, or foodborne pathogens [17,18,19,20]. The QMRA model uses measures of microorganisms in the environment as inputs to calculate associated risks as outputs. QMRA studies can be used to estimate the risk of illness and/or infection due to the exposures from various doses of waterborne pathogens [21,22,23]. QMRA has been widely used for microbial risk assessment from water (natural recreational water, wastewater treatment plants, rivers, seawater, and wastewater) and food sources, in both community and occupational settings [17,18,20,21,22]. To the best of our knowledge, this is first of study to estimate the risks posed by microbial contamination in drinking water, in school settings.

The specific objectives of this study include the characterization of water-supply conditions in primary schools in Sindh, Pakistan, and the application of QMRA to estimate the risk of infections in children attending those schools. These findings will help policymakers to develop water-safety plans.

## 2. Materials and Methods 

### 2.1. Study Area (Sindh)

**Figure 1:**
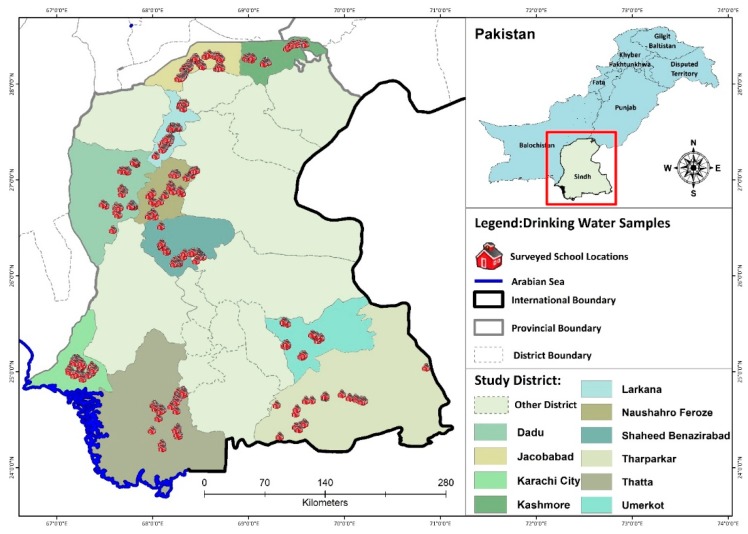
Surveyed school locations in different districts of Sindh.

Pakistan is administratively divided into four provinces. Sindh province is the second largest province, with a population of around 4.8 million, and is located in the southeast of the country. Sindh province has three climatic regions (units), north, south, and central, and each region is subdivided into districts. Sindh has 29 districts. The main source of drinking water in Central Sindh is groundwater; in North Sindh, it is groundwater and surface water; and South Sindh mainly depends on surface water (Figure 1). The districts in South Sindh, especially Karachi and Sujawal, are near the sea, therefore, groundwater is too salty and cannot be utilized for drinking water purposes without treatment, so surface water is the main source of drinking water in these both districts [15,24]. A thorough water, sanitation, and hygiene (WASH) survey, along with microbial water-quality analysis, was carried out in all the selected schools.

### 2.2. Sampling

A total of 42,900 schools are registered with the Government Education Department of Sindh [25], and a sample size of 425 schools was selected, using multistage random-sampling techniques. The sampling scheme produces a random sample, representative of the whole of Sindh province (Table 1). In the first phase, 10 districts were randomly selected, with a probability of selection proportionate to size, and then in the second stage, primary schools were randomly sampled from each district, to accomplish drinking-water sampling from 425 schools. All water samples were taken in the same season, between November and January. The schools were assessed using the WHO standard WASH assessment tool [26]. The headmaster of each school was interviewed, and drinking-water samples and structured observations of WASH were collected. ODK Collect Android Software was used to collect survey data [27]. Structured observations of water facilities were carried out, to assess the availability, accessibility, and functionality of water facilities. The questionnaire was translated into both English and Sindhi (local language), to ensure ease of use. The survey was pre-tested among six schools, to evaluate clarity of the translated questions, the appropriateness of the wordings based on the cultural and belief, and the duration of time taken to answer the questionnaire. 

### 2.3. Microbial Contamination of Drinking-Water Sources

Microbial risk assessment was carried out. We first collected drinking-water samples from selected primary schools. The most commonly found bacteria, including *Escherichia coli*, *Salmonella* spp., *Vibrio cholerae*, and *Shigella* spp., were considered for QMRA tool [28,29]. QMRA involves estimating the risk of infection or illness due to exposure to pathogenic microorganisms [28]. It consists of (1) hazard identification, (2) dose–response assessment, (3) exposure assessment, and (4) risk characterization [28]. Our primary data include identification of *E. coli, Salmonella* spp., *V. cholerae*, and *Shigella* spp. *E. coli* is considered an indicator organism and is used in the present study, to estimate the risk of two other commonly reported pathogenic contaminants, i.e., *Campylobacter* and *Rotavirus*, in the drinking-water sources [29,30,31]. The data regarding exposed population, frequency of water consumption (V = 1 Liter per day for children), and exposure pathways were estimated from previous studies [29,30].

### 2.4. Hazard Identification

Measures of *E. coli*, *Salmonella* spp., *Shigella* spp., and *V. cholerae* were used to directly estimate students’ exposures to bacterial contaminants through drinking water available in primary schools. Furthermore, *Campylobacter* and *Rotavirus* were estimated on the basis of indicator organism. Pathogenic strains of *E. coli* are major hazards for public health [30]. The literature revealed that the 8% of the total *E. coli* is pathogenic, so the *E. coli* dose was multiplied by 0.08, in order to estimate the dose of pathogenic *E. coli*, as has been done elsewhere [28,29]. *Campylobacter* and *Rotavirus* were also predicted, using the *E. coli* to pathogenic ratio [19,29]. From each school’s drinking-water source at the point of use, 1500 mL of drinking water was collected in a sterile bag. The collected sample was preserved in an ice box, at a temperature of approximately 4 °C, and then the samples were transported to the ISO certified national laboratory, for the detection of selected pathogenic bacteria from drinking-water samples.

### 2.5. Isolation and Identification of Bacteria in Drinking Water 

The membrane filtration method and selective agar media were used for the detection of *E. coli* (TBX agar Sigma), *V. cholerae* (*Cholera* Medium TCBS Oxoid England), *Shigella*, and *Salmonella* spp. (X.L.D. Agar Oxoid England). The standard membrane-filtration method was used for the detection of *E. coli*, *V. cholerae*, *Shigella*, and *Salmonella* spp. Every bacterium isolated separately, using 100 mL water samples, was filtered through membrane filtration, and the filter was placed on the selective agar plates, for incubation, for 22 to 24 h. After incubation, the number of colonies was counted on selective agars plates and reported in colony-forming unit (CFU/100 mL); all the samples were analyzed in replicate, and results were recorded in average, using standard drinking-water testing techniques [29].

### 2.6. Dose–Response Assessment

The dose–response (DR) assessment characterizes the relationship between the number of pathogens ingested (dose) and the likelihood of occurrence of an adverse consequence, in terms of infection, illness, or death. A β-Poisson dose–response model is shown in Equation (1): [28]

(1) $$\frac{Pinf}{day}=1-\left[1+\left(\frac{d}{N50}\right)\left(2^{\frac{1}{\propto }}-1\right)\right]$$

where d is the dose, N50 is the dose corresponding to the median response, and *alpha* is a parameter of the distribution. Our models’ parameter assumptions are shown in Table 2.

### 2.7. Exposure through Drinking-Water Sources 

The average number of days per year when water was consumed from a source was 300 days; the remaining days were vacations and/or public holidays. The consumed volumes was 1 liter per day per child, and they were estimated as done in previous studies [29,30] and based on our survey data. 

### 2.8. Risk Characterization 

For risk characterization, the probability of annual infection and probability of illness are determined by using a standard equation [20]. The annual probability of infection is calculated via Equation (2):

(2) $$P\;inf\;annual=1-{\left[1-Pinf/day\right]}^{n}$$

where the probability infection per day (Pinf/day) was obtained from Equation (1), above, and n is the number of exposure days in a year. The probability of illness was calculated by Equation (3):

(3) Pill=P infannual ×Pill/inf

where Pill/inf is the probability of illness per infection, and P inf annual is defined in Equation (2).

## 3. Results

### 3.1. Characterization of Water Samples

Drinking-water samples from the point of use were collected in all 425 primary schools. As described earlier, the most common sources of drinking water were groundwater in Central Sindh, groundwater and surface water in North Sindh, and surface water in South Sindh (Table 1). The sources of water at school were mainly groundwater or dug-well (62%) and surface water supplied through pipes and water cans/plastic bottles (38%). Year-round availability of water from the main source was reported by one-third of schools (33.4%). Onsite water-treatment systems were available at 20.2% of the schools, in which filtration was used as the main method (commercial filters, including carbon, limestone, and fiber thread), followed by boiling, chlorination, and ultraviolet disinfection. None of the schools had tested the drinking water’s quality during the past two years.

Other data we collected indicated that only one-third of the schools, mainly private schools, had water-storage capacity, such as an overhead or underground tank. None of these tanks had been cleaned in the past six months. Using the United Nations’ (UN) joint monitoring program (JMP) WASH’s definitions for schools [34], water-service levels at these selected schools were assessed, and 7% of schools had advanced service levels, 57.4% had basic, 19.8% had limited, and 16% of schools had no service at all.

### 3.2. Microbial Quality of Drinking Water

Our study showed that, across all schools, 49% of the drinking-water samples were contaminated with *E. coli*, 54% with *Salmonella* spp., 49% with *V. cholerae*, and 63% were contaminated with *Shigella* (Table 3). *E. coli* contamination in drinking water was highest in the Karachi district, followed by S. Benazirabad and Umerkot. *Salmonella* spp. contamination was higher in Larkana, Naushahro Feroze, and Umerkot than the other districts. Additionally, water samples from Naushahro Feroze exhibited more *V. cholerae* contamination than the other districts. 

Water contamination was worse in South Sindh, where surface water is an important source of drinking water. Water was highly contaminated with *E. coli* (Umerkot 68.9%, Karachi 60%, and Sujawal 57.5%). There was also a high prevalence of *Salmonella* spp. in the drinking-water samples of Larkana (69%), Umerkot (64.4%), and N. Feroze (64.4%). The prevalence of *Salmonella* spp. was comparatively lower in Northern Sindh (Kashmore 50.1% and Jacobabad 47.5%). *Vibrio cholerae* contamination was higher in the drinking-water samples of Central Sindh (N. Feroze 93.3%, Umerkot 60%) and lower in the Northern Sindh (Dadu 31% and Jacobabad 22.5%). Overall, our study revealed that the there is a high prevalence of *V. cholerae* and *Shigella* in the drinking-water samples. The prevalence of *Shigella* (Table 3) was highest in the southern districts of Sindh (Karachi 90% and N. Feroze 82.2%) and lowest in the northern districts (Larkana 45.2%, Dadu 54.8%, and Kashmore 52.4%; Table 3). 

The average number of bacteria ingested was calculated; the highest ingestion doses per day were for *V. cholerae,* followed by *Shigella* and then *Campylobacter* (Table 4). The number of *E. coli* ingested per day was the highest in Jacobabad and Karachi and was the lowest in the Kashmore and Larkana districts. *Shigella* ingestion was the highest in the central region of Sindh (Naushahro Feroze, Dadu) and the lowest in S. Benazirabad.

**Figure 2:**
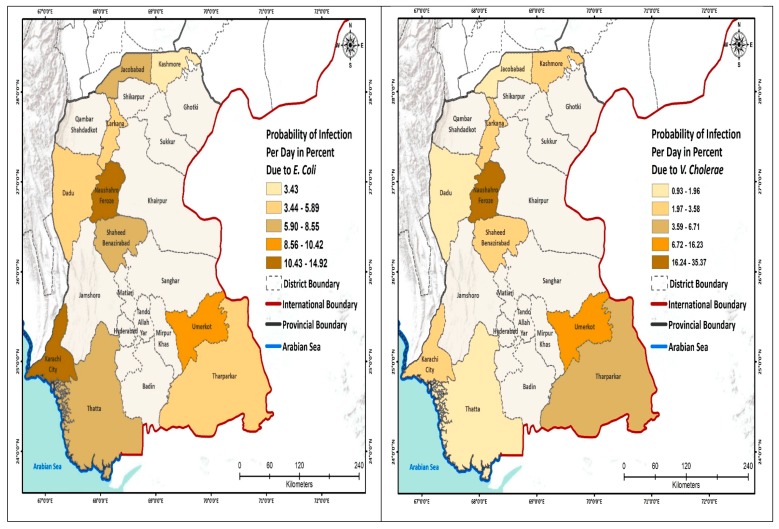

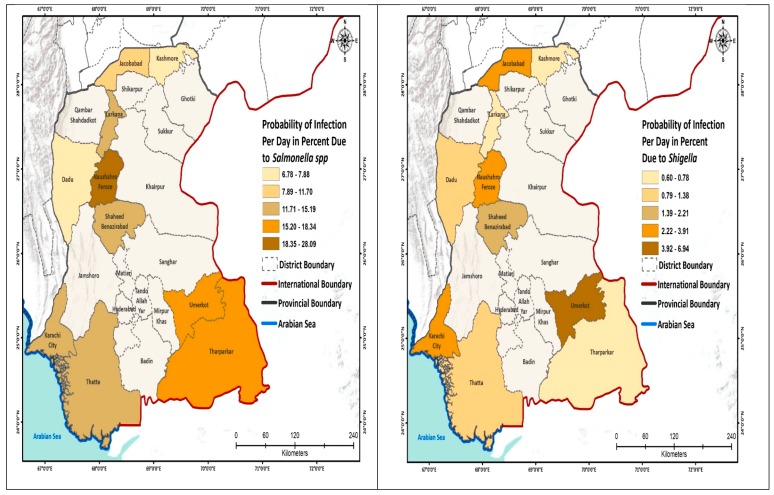

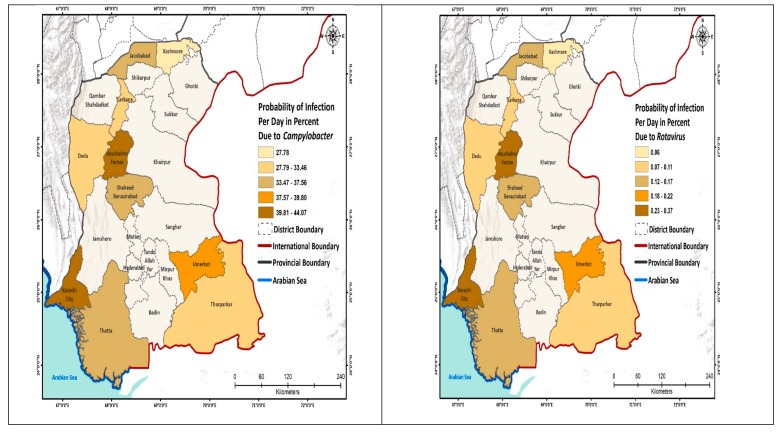
Probability of infection (P_inf_) to the schoolchildren per day.

We further analyzed the probability of infection to schoolchildren per day and show this by geographic area (Figure 2; Appendix A Table A1). The highest risk due to the *E. coli* was estimated for the schools located in South Sindh (Karachi 14.9%), using mainly surface water for drinking purpose, whereas the lowest risk of infection was calculated for the schools of North Sindh, mainly using groundwater. The risk from *Salmonella* spp. was the highest in Naushahro Feroze (28.1%), where children were exposed to a mix of surface and groundwater, while the lowest risk of infection was 6.8% in the Kashmore district. The risk of infection due to the *V. cholerae* was the highest in the schools located in Central Sindh that used a mix of water sources; these included Naushahro Feroze 35% and Jacobabad (0.93%). The risk of infection from *Shigella* was highest in Naushahro Feroze (6.9%) and the lowest in Kashmore (0.60%). The daily risk to *Rotavirus* was very low. The risk due to the *Campylobacter* was higher in the schools in the south, especially in Karachi, whereas the risk was comparatively low in the northern districts (Figure 2).

We estimated the probability of infection per year for children in primary schools (Table 5). Across all districts, drinking water from primary schools was estimated to lead to an extremely high risk (>94%) of infection by each of the bacteria assessed; the probability of infection from *Rotavirus* was only 23.4%. The risk of illness due to the bacterial infection was also high as compared to the *Rotavirus*. About 70% of children were at risk of illness due to *Campylobacter*, 35% were at risk of illness due to *E. coli*, 33% due to *Shigella*, 45% due *to Salmonella* spp., and 20% of children were at risk of illness due to *V. cholerae*. The risk of illness due to each of the bacteria had little variation within districts, whereas there was some variation by district for illness by *Rotavirus.* Schoolchildren in Karachi were estimated to have more risk of *Rotavirus*-related illness (22.6%), as compared to children in the Larkana (3%) and Kashmore (3%) districts.

The Pearson correlation analysis showed high correlations between various pathogens (Table 6). The strongest associations were between *E. coli* and *Campylobacter*; between *E. coli* and *Rotavirus*; between *V. cholerae* and *Salmonella*; between *V. cholerae* and *Shigella*; between *Shigella* and *Salmonella* spp.; and between *Campylobacter* and *Rotavirus.*

## 4. Discussion

Our study characterized water, sanitation, and hygiene (WASH) conditions in 425 primary schools of Sindh, Pakistan, and estimated the risk of waterborne infections in schoolchildren. Overall, the risk of illness due to the bacterial infection (*E. coli*, *Salmonella* spp., *V. cholerae, Shigella*, and *Campylobacter)* was high.

We observed that, on average, almost half (49%) of the drinking-water samples were contaminated with *E. coli*, 54% with *Salmonella* spp., 49% with *V. cholerae*, and 63% with *Shigella*. South Sindh mainly depends on surface water for drinking water, and that water appears to be highly contaminated with *E. coli* (Umerkot 68.9%, Karachi 60%, and Sujawal 57.5%). The high risks of infections that we found in the present study are congruent with other available studies of drinking water in Pakistan [15,24,35]. The latest Pakistan demographic and health survey in the community settings of the study area reported a high prevalence of waterborne diseases, such as diarrhea, dysentery, cholera, and typhoid [36], which aligns with the high-risk pathogens we found in our study. Recently, a prestigious hospital of the study area has reported the emergence of the first large-scale spread of an extensively drug-resistant (XDR) *Salmonella typhi*, exhibiting resistance to Trimethoprim–sulfamethoxazole, ampicillin, chloramphenicol, fluoroquinolones, and third-generation cephalosporins [37]. The ongoing outbreak has resulted in more than 300 cases, including 187 pediatric cases since November 2016 [37]. The WHO maintained that the Sindh province of Pakistan is under high risk of acquiring XDR *Salmonella* spp., because of poor access to clean water, as well as unimproved sanitation and hygiene [38]. In our study, there was a higher prevalence of *Salmonella* spp. in the drinking-water samples from South and Central Sindh districts, especially in Umerkot (64.4%) and N. Feroze (64.4%). The prevalence of *Salmonella* spp. was comparatively lower in Northern Sindh (Kashmore 50% and Jacobabad 37%). 

The global burden of *V. cholerae* and *Shigella* is mostly unidentified, because the majority of the cases go unreported [7,10]. Poor reporting can be ascribed to economic, social, and political disincentives for reporting, as well as the inadequate capacity of laboratories and epidemiological surveillance in schools of low resource countries [39,40,41]. In our study, *V. cholerae* contamination was higher in the drinking-water samples of Central Sindh, and lower in the Northern Sindh. Almost half of the schools in the study area possess unimproved sanitation system [9]. Poor sanitation poses frequent risks of *cholera* and *shigellosis* [42]. Our study findings also revealed a high prevalence of *V. cholerae* and *Shigella* pathogens in the analyzed drinking-water sources. *Shigella* prevalence was highest in the southern districts of Sindh and lowest in the northern districts.

School-going children in Karachi were estimated to have a higher risk of *Rotavirus*-related illness (22.6%) than children in the N. Feroze district (10.2%) and lowest in Umerkot and Larkana districts (3%), where groundwater was the primary source of drinking water. Several studies in the region have attributed *Rotavirus* as an important cause of illness in children. A study in a tertiary-level hospital in Sindh Pakistan found *Rotavirus* among 63% of children presenting with diarrhea [43]. The Indian subcontinent (Pakistan, Bangladesh, and India) attributes three-fourths of the deaths to *Rotavirus*, which is considered the most common cause of severe diarrhea among young children [44].

Waterborne *Campylobacter* infections are associated with recurrent diarrhea in children, which ultimately affect children’s school performance [45]. In our study, the risk of *Campylobacter* infection was higher among the schoolchildren in the south and lower in children studying in the northern region. The south region is located at the tail end of the Indus River, where surface water is the main source of drinking water. The waterborne diseases among children in that region are reported quite high [7,15]. There can be multiple reasons for source-water contamination, including mixing up of industrial or household waste into the surface water without treatment. Additionally, a poorly functioning water-treatment system could also be a problem. Non-separation of waste and water supply line result in contamination of the water-system network [29]. The delivery of improved water at schools can contribute to the improved health and educational performance. Further, water helps to maintain environmental and personal hygiene, decrease chances of dehydration at schools, and ultimately contribute to improved cognitive abilities [27,46].

The geographical differences in pathogen-related health risks between North and South Sindh were likely due to different types of water sources. Across all sites, generally schools that used surface water had a higher risk of infection. This is similar to many other studies that have found the highest burden of waterborne diseases in a low-income population, using surface water as a main source of drinking [47].

Surface water in places with poor infrastructure can create exposure to contamination through open drainage channels, unprotected tertiary drains, and contaminated soil [47,48]. Diseases related to inadequate water, sanitation, and hygiene carries a huge burden of disease in low-income countries. Schools situated in rural settings often lack drinking-water, sanitation, and hand-washing facilities; even where such facilities exist, they are often inadequate in both quantity and quality. Schools deprived of improved water sanitation and hygiene conditions pose a high risk of disease transmission by intense levels of person-to-person contact [34,49].

There were several limitations in our study. Firstly, students might not be drinking from the school water sources, but instead drink from their personal water bottles, brought from home. Secondly, we only studied six pathogens, based on our limited resources; however, there are other waterborne pathogens which may be considered in future studies. Further, our estimates of pathogenic *E. coli*, of *Campylobacter*, and of *Rotavirus* were based on predicted doses, using assumptions from the literature and using our measure of total *E. coli*. Thirdly, our results have not taken into consideration the uncertainty of the risk of our estimates (e.g., like Monte-Carlo analysis would do). Finally, we only collected a single sample of water from each school, whereas water quality may vary seasonally.

## 5. Conclusions

QMRA is a useful tool that is widely used in community settings, to predict the probability of infection and illness in large populations due to exposure to microbiologically unsafe water. This is the first study conducted in school settings that used the QMRA tool to estimate the risks of waterborne diseases through drinking-water sources. The study findings revealed that drinking water in each region presents varying degrees of health risk. Our QMRA results will help to inform the water-quality situation, health-risk poses to the study population, and dire need for management plans in schools of Sindh. Improved access to safe water and sanitation are critical to halt the progression of waterborne diseases. For improving drinking-water quality, we recommend periodic water-quality monitoring and, where necessary, the use of simple, low-cost, and sustainable drinking-water treatment methods, such as boiling, solar disinfection, and chlorination. 

## Figures and Tables

**Table 1:** Details about the sampled districts, schools, and sources of the water samples.

Location	Districts	N Primary Schools	Sources of Drinking Water
**North Sindh**	Larkana	42	Ground (88%), Surface (12%)
	Jacobabad	40	Ground (67%), Surface (33%)
	Kashmore	42	Ground (83%), Surface (16%)
**Central Sindh**	S. Benazirabad	47	Ground (87%), Surface (13%)
	Dadu	42	Ground (83%), Surface (17)
	Naushahro Feroze	45	Ground (94%), Surface (6%)
**South Sindh**	Tharparkar	42	Surface (55%), Ground (45%)
	Sujawal	40	Surface (85%), Ground (15%)
	Karachi	40	Surface (98%) Ground (2%)
	Umerkot	45	Ground (58%), Surface (42%)
**Total samples**		425	

**Table 2:** Dose–response parameters.

Organisms	Parameters	Type of Model	Reference
*Escherichia coli*	α = 0.2099 N50 = 1120 PDi = 0.35	β-Poisson model	[28,29,30,32]
Campylobacter *	α = 0.145 N50 = 896 PDi = 0.7	β-Poisson model	[29,32]
Rotavirus *	α = 0.2531 N50 = 6.17 PDi = 0.5	β-Poisson model	[28,29]
*Salmonella* spp.	α = 0.21 *β* ** = 49.78 N50 = 1.11 × 10^6^ PDi = 0.45	β-Poisson model	[33]
*Shigella* spp.	*α =* 0.265 β ** =1480 PDi = 0.35	β-Poisson model	[33]
*Vibrio cholerae*	*α =* 0.250 β ** = 243 PDi = 0.2	β-Poisson model	[33]

* Predicted based on assumptions from the literature, using total measured *E. coli*. ** N50 can be reparametrized in terms of β [29].

**Table 3:** Frequency of microbial contamination.

Districts	N Schools	% with Contaminated Water Sources			
		*E. Coli*	*Salmonellae* spp.	*V. Cholerae*	*Shigella*
Dadu	42	50.0	47.6	31.0	54.8
Jacobabad	40	42.5	37.5	22.5	67.5
Karachi	40	60.0	42.5	27.5	90.0
Larkana	42	21.4	69.0	57.1	45.2
S. Benazirabad	47	57.4	55.3	55.3	76.6
Sujawal	40	57.5	47.5	30.0	57.5
Tharparkar	42	23.8	57.1	59.5	52.4
Naushahro Feroze	45	64.4	64.4	93.3	82.2
Umerkot	45	68.9	64.4	60.0	55.6
Kashmore	42	45.2	50.0	57.1	52.4
**Overall**	425	49.4	53.9	50.1	63.5

**Table 4:** Average bacterial ingestion by children.

Districts	Average Bacterial Ingestion Colony Forming Unit (CFU)/Day					
	*E. Coli*	*Salmonella* spp.	*V. Cholerae*	*Shigella*	*Campylobacter*	*Rotavirus*
Dadu	0.8476 × 10^1^	2.381 × 10^1^	1.3095 × 10^1^	8.2619 × 10^1^	6.9928 × 10^1^	10.5 × 10^−4^
Jacobabad	0.4375 × 10^1^	4.025 × 10^1^	0.925 × 10^1^	1.3475 × 10^2^	3.6135 × 10^1^	54.7 × 10^−5^
Karachi	2.916 × 10^1^	5.4 × 10^1^	2.425 × 10^1^	2.49.5 × 10^2^	2.405 × 10^2^	36.01 × 10^−4^
Larkana	0.295 × 10^1^	5.9286 × 10^1^	2.7143 × 10^1^	3.7381 × 10^1^	2.4357 × 10^1^	36.9 × 10^−5^
S. Benazirabad	0.925 × 10^1^	5.9167 × 10^1^	3.8125 × 10^1^	1.01667 × 10^2^	7.631 × 10^1^	11.56 ×10^−4^
Sujawal	0.518 × 10^1^	5.55 × 10^1^	2.0 × 10^1^	6.025 × 10^1^	4.273 × 10^1^	64.7 × 10^−5^
Tharparkar	0.468 × 10^1^	7.8095 × 10^1^	7.7857 × 10^1^	4.619 × 10^1^	3.868 × 10^1^	58.5 × 10^−5^
Naushahro Feroze	1.109 × 10^1^	1.89556 × 10^2^	1.149556 × 10^3^	4.80 × 10^2^	9.154 × 10^1^	13.8 × 10^−5^
Umerkot	1.484 × 10^1^	8.0889 × 10^1^	2.5044 × 10^2^	1.9022 × 10^2^	1.222 × 10^2^	18.5 × 10^−4^
Kashmore	0.331 × 10^1^	1.976 × 10^1^	3.6429 × 10^1^	3.5238 × 10^1^	2.734 × 10^1^	41.4 × 10^−5^

Colony Forming Unit (CFU)/day = C*V; C = mean concentration of bacteria; V = 1 liter per day for children [29,30]. Probability of bacterial ingestion per day based on beta-Poisson dose-response model [28,30]. Parameter values listed in Table 2.

**Table 5:** Probability of infection per year and probability of illness per year among schoolchildren, due to school drinking-water exposure.

District	Probability of Infection per Year (%)						Probability of Illness per Year (%)					
	*E. Coli*	*Salmonella* spp.	*V. Cholerae*	*Shigella*	*Campylobacter*	*Rotavirus*	*E. Coli*	*Salmonella* spp.	*V. Cholerae*	*Shigella*	*Campylobacter*	*Rotavirus*
Dadu	100.0	100.0	97.3	97.8	100.0	1.61	35.0	45.0	19.5	34.2	70.0	8.0
Jacobabad	99.6	100.0	92.3	99.8	100.0	8.6	34.9	45.0	18.5	34.9	70.0	4.3
Karachi	100.0	100.0	99.9	100.0	100.0	45.1	35.0	45.0	20.0	35.0	70.0	22.6.
Larkana	97.9	100.0	99.9	82.9	100.0	5.9	34.3	45.0	20.0	29.0	70.0	3.0
S. Benazirabad	100.0	100.0	100.0	99.1	100.0	17.4	35.0	45.0	20.0	34.7	70.0	8.7
Sujawal	99.9	100.0	99.6	94.0	100.0	10.1	35.0	45.0	19.9	32.9	70.0	5.1
Tharparkar	99.8	100.0	100.0	88.4	100.0	92.0	34.9	45.0	20.0	31.0	70.0	4.6
Naushahro Feroze	100.0	100.0	100.0	100.0	100.0	20.5	35.0	45.0	20.0	35.0	70.0	10.2
Umerkot	100.0	100.0	100.0	100.0	100.0	26.4	35.0	45.0	20.0	35.0	70.0	13.0
Kashmore	98.7	100.0	100.0	80.9	100.0	6.6	34.5	45.0	20.0	28.3	70.0	3.3
**Overall**	99.6	100.0	98.9	94.3	100.0	23.4	34.8	45.0	19.8	33.0	70.0	6.7

**Table 6:** Pearson correlation between pathogens.

	*E. Coli*	*V. Cholerae*	*Shigella*	*Campylobacter*	*Rotavirus*	*Salmonella* spp.
*E. coli*	1					
*V. Cholerae*	0.118	1				
*Shigella*	0.525	0.878 *	1			
*Campylobacter*	1.000 *	0.118	0.525	1		
*Rotavirus*	1.000 *	0.118	0.525	1.000 *	1	
*Salmonella* spp.	0.156	0.942 *	0.833 **	0.156	0.156	1

* Correlation significant at the 0.01 level (two-tailed).

## Appendix A

**Table A1:** Probability of infection per day (Pinf).

Districts of Sindh Selected for This Study	P_inf_ *E. Coli*/Day	P_inf_ *S. Typhi*/Day	P_inf_ *V. Cholera*	P_inf_ *Shigella*	P_inf_ *Campylobacter*	P_inf_ *Rotavirus*
Dadu	0.0372	0.0788	0.013	0.0138	0.2861	3.2 × 10^−4^
Jacobabad	0.0202	0.117	0.0093	0.0221	0.2243	1.6 × 10^−4^
Karachi	0.1033	0.143	0.0235	0.0391	0.3969	10.9 × 10^−4^
Larkana	0.0139	0.1519	0.0261	0.0064	0.1881	1.1 × 10^−4^
Nawabshah	0.0402	0.1517	0.0358	0.0169	0.2942	3.5 × 10^−4^
Sujawal	0.0236	0.1455	0.0196	0.0102	0.2399	1.9 × 10^−4^
Tharparkar	0.0215	0.1797	0.0671	0.0078	0.2305	1.8 × 10^−4^
Noushehro Feroze	0.0472	0.2809	0.3537	0.0694	0.3111	6.9 × 10^−4^
Umerkot	0.0605	0.1834	0.1623	0.0305	0.3377	5.6 × 10^−4^
Kashmore	0.0155	0.0678	0.0343	0.006	0.1986	1.2 × 10^−4^
